# The impacts of training pathways and experiences during intern year on doctor emigration from Ireland

**DOI:** 10.1186/s12960-019-0407-z

**Published:** 2019-11-06

**Authors:** Frances Cronin, Nicholas Clarke, Louise Hendrick, Ronan Conroy, Ruairi Brugha

**Affiliations:** 1 RCSI Division of Population Health Sciences, Royal College of Surgeons in Ireland, Lower Mercer Street, Dublin 2, Ireland; 2 School of Psychology, Dublin City University, Glasnevin, Dublin 9, Ireland; 3 National Doctors Training and Planning, Health Service Executive, Sancton Wood Building, Heuston South Quarter, St John’s Road West, Dublin 8, Ireland

**Keywords:** Medical migration, Medical workforce planning

## Abstract

**Background:**

Emigration of domestically-trained health professionals is widespread, including in Ireland which has the highest rate of medical graduates in the OECD. Ireland’s failure to retain graduates necessitates high levels of international recruitment. This study aimed to identify factors associated with recently graduated doctors’ intention to migrate, focusing on their work experiences during the mandatory post-graduation year, their wellbeing, and their perceptions of postgraduate training in Ireland.

**Methods:**

A baseline survey was administered online to all final year students in Ireland’s six medical schools. A subsequent sweep surveyed those who consented to follow-up (*n* = 483) during the final month of first year of practice.

**Results:**

Of the 232 respondents (48% response rate), 210 (94%) were Irish passport holders. Of these, only 36% intended to remain in Ireland after their internship, 57% intended to leave but return later, and 7% intended to leave permanently. A strong predictor of intention was study pathway: 60% of Graduate Entry Medicine (GEM) graduates and 25% of Direct Entry Medicine (DEM) graduates intended to remain in Ireland. Equal proportions intended to leave permanently (8% DEM, 6% GEM). Being a GEM graduate significantly reduced the likelihood of leaving to return (relative risk ratio (RRR) 0.20, 95% confidence interval (CI) (0.11–0.39), *p* < 0.001).

When adjusted for study pathway, a negative experience as an intern increased the likelihood of leaving to return (RRR 1.16 CI (1.00–1.34), *p* = 0.043) and leaving permanently (1.54 (1.15–2.04), *p* = 0.003). Similarly, experience of callousness was associated with leaving to return (1.23 (1.03–1.46), *p* = 0.023) and leaving permanently (1.77 (1.24–2.53), *p* = 0.002), as was burnout with leaving permanently (1.57 (1.08–2.27), *p* = 0.017). Those planning to specialise in Medicine versus General Practice were more likely to leave and return (3.01 (1.09–8.34), *p* = 0.034). Those with negative perceptions of training in Ireland were more likely to leave and return (1.16 (1.01–1.34), *p* = 0.037); a positive perception reduced the likelihood of leaving permanently (0.50 (0.26–0.94), *p* = 0.032).

**Conclusions:**

Increasing GEM training places might improve Ireland’s retention of domestically-trained doctors, reducing reliance on non-EU-trained doctors. However, improvements in the working experiences, perceptions of training, and protection of wellbeing are essential for retaining this highly sought-after and geographically mobile cohort.

## Background

The deleterious effects of the emigration of domestically-trained medical personnel on a country’s health workforce is a global phenomenon, no longer confined to low- or middle-income countries [1, 2]. Medical graduates are an expensively trained, highly sought-after and geographically mobile cohort [3, 4]: The United Kingdom (UK) reports an estimated 60% of recent medical graduates intending to migrate [5], Portugal reports 55% [6], and Germany 30% [7]. In Romania, almost all (85%) medical students surveyed between 2013 and 2015 reported that they intended to emigrate on graduation [8]. Increasingly, countries are losing their home-trained doctors and investing in recruiting and incorporating immigrant doctors into their healthcare systems, without knowing how long they will remain [9–12].

This phenomenon is seen in Ireland’s healthcare system today. Currently, perhaps as a result of an historic reliance by Irish medical schools on non-EU student fees to subsidise the delivery of their medical programmes [13, 14], Ireland has the highest number of medical graduates per 100,000 population of the Organisation for Economic Co-operation and Development (OECD) countries [15]. At 24.4 medical graduates per 100,000, Ireland far outstrips Australia (15.9), the United Kingdom (12.9), Germany (11.7), and Canada (7.9). Despite this high production, Ireland has a similar number of practising doctors^1^, at 3.1 per 1000, to Germany (4.2 per 1,000), Australia (3.6), the United Kingdom (2.8), and Canada (2.7) [15]. Furthermore, the proportion of Ireland’s practising doctors recruited internationally continues to rise, from 13.4% in 2000 to 33.4% in 2010 [11], to 42% in 2017 [16]. This loss of home-trained doctors, and the resulting reliance on non-EU-trained doctors, undermines compliance with the WHO Global Code on the International Recruitment of Health Personnel [17]. The Code commits signatories (of which Ireland is one) to discouraging active recruitment of health personnel from developing countries that face shortages of their own. The continuing high rates of doctor emigration are a cause of concern to Irish healthcare planners [14] and is a topic for much medical workforce research [18–21].

A number of studies of Irish doctors have sought to estimate and analyse emigration intentions and underlying factors that might be compelling doctors to leave Ireland. Findings suggest that experiences of poor working conditions and perceptions of superior conditions in the destination country act as factors that ‘push’ and ‘pull’ doctors to emigrate from Ireland [18, 22, 23]. As an example, in a survey of 523 doctors undertaking postgraduate training in Ireland in 2016, views on work-life balance and perceived quality of training discriminated between those intending to leave and those planning to continue their careers in Ireland [18]. Similarly, recent qualitative research identified long working hours and uncertain career progression in Ireland as contributory factors to the decision to migrate [20, 21]. And recently published research, from a 2014 national study of wellbeing among hospital doctors (*n* = 1,749) in Ireland, reported that more than one in three met the criterion for burnout [24]. Together, these findings, set within the Irish context, echo research on the reasons why doctors emigrate from other OECD countries including the United Kingdom [5], Germany [7], and New Zealand [25, 26].

However, in some countries, there may be a cohort of medical graduates that migrates solely to complete specialist training abroad, with a view to returning to their home country to take up specialist consultancy posts [20, 27]. Historically, this ‘circular’ pattern of migration has been accepted as a route to securing consultant posts in Ireland: indeed when competing for posts against domestically-trained doctors, the returning internationally-trained doctor has been considered by many to be viewed preferentially by interviewing panels [20]. However, in countries with a history of circular migration such as Ireland, in more recent times, an increasing proportion of doctors who emigrated with the intention of returning to their homeland has decided not to return [20, 28, 29]. For example, one study traced almost 300 UK-trained doctors, who, 10 years after their graduation, were registered to practise in New Zealand. Of these doctors, only 30% had originally intended to emigrate permanently; however, 10 years later, 89% were intending to stay in New Zealand permanently [25]. Similarly, a 2014 study surveyed 307 Irish-trained doctors who had been working in Australia since 2008. When leaving Ireland, only 10% of the group had intended to stay in Australia permanently and 50% of them intended to return to Ireland to practise medicine. When surveyed up to 5 years later, 34% intended to stay in Australia permanently, and only 25% planned to return to Ireland [28].

The aim of this paper is to identify and examine the factors associated with an intention to migrate among early-career doctors. Factors of interest include the hospital working experience during internship and the graduate doctors’ perceptions of post-graduate training in Ireland. In particular, the paper reports on whether the emotional exhaustion (burnout) and feelings of depersonalization (callousness), reported in more experienced hospital doctors, had already manifested within 12 months of graduates starting work, and whether or not these factors were associated with an intention to emigrate.

## Methods

This research examined factors—both personal and professional—that predict medical graduates’ intention to migrate and used cross-sectional data from the intern wave of the *MedTrack* Study, which is a prospective, longitudinal, observational study comprising all Irish/European Union final year medical students who graduated in Ireland in 2017. In Ireland, there are two study pathways available to medical students: a 5–6-year Direct Entry Medicine (DEM) undergraduate programme or a 4-year Graduate Entry Medicine (GEM) programme. The GEM programme was proposed in 2006 to boost domestic production of doctors [30] and is available in four of the six Irish medical schools. GEM applicants hold a previous degree in another discipline. On graduation, both DEM and GEM students must complete a mandatory 1-year internship in accredited Irish hospitals to be awarded their ‘Certificate of Experience’, whereupon the Medical Council of Ireland then allows full registration as medical practitioners.

As part of a study on career choice, which asked respondents about their intended speciality during the final year of undergraduate training (paper in preparation), a baseline sweep was administered on-line between November 2016 and February 2017 to all final year students in Ireland’s six medical schools. Respondents were asked to provide an email address for follow-up purposes. The intern sweep was administered during June 2018—the final month of the mandatory internship year.

Intern survey items were informed by recent qualitative and quantitative research on doctors working in Ireland [18, 21, 23, 31], together with international research on career choices by medical students [32, 33]. Items were selected to identify factors, including personal attributes, and career experiences and perceptions, that might affect an early-career doctor’s intention to migrate. Specifically, the tool measured their satisfaction with different dimensions of training and working conditions during their internship year, and whether early dissatisfaction plays a role in an early-career doctor’s intention to migrate.

Questions were developed to specifically address issues known to be of concern to the Medical Council (the regulatory body for doctors in Ireland involved in medical education and training of doctors) regarding experience during the intern year, together with perspectives on and perceptions of postgraduate training in Ireland [34]. Working conditions during internship were measured by presenting items regarding, e.g. supervision and non-core task allocation, with the option to rate these as ‘High/Acceptable/Low’. The prevalence of emotional exhaustion (burnout) and depersonalisation (callousness) experienced in the previous 12 months as an intern was captured using validated, single-item measures [35, 36]. Respondents’ perceptions of medical training in Ireland were evaluated by rating as ‘True/False/Don’t know’ statements derived from previous research [18, 23] and from a national strategy document proposing measures to improve the retention of doctors in training posts [14].

Respondents identified their first choice of long-term specialty, and the initial 18 categories were collapsed to correspond with categories employed previously in the literature. The survey also contained items measuring current level of debt (as a result of study), and medical school study pathway (DEM or GEM).

Respondents were asked to indicate their intention to migrate by selecting one of three options: ‘Remain in Ireland to practice medicine’ (remain); ‘Go abroad to practice medicine, but return to Ireland to continue my medical career’ (leave but return); or ‘Go abroad to practice medicine and not return to Ireland’ (leave permanently).

Ethics approval was obtained from the Royal College of Surgeons in Ireland (references REC1252 and REC1252b). Informed consent was obtained from all respondents, fulfilling General Data Protection Regulations requirements [38].

### Statistical analysis

Analysis was undertaken using StataIC, Version 15. Multinomial logistic regression analysis of doctors intending to migrate compared doctors intending to ‘leave but return’, and ‘leave permanently’, with doctors intending to ‘remain’ as the comparison group. Factors collapsed and/or dichotomised were level of debt (€10K and over); age (27 years and over), and those with negative experiences versus those with ‘positive’ or ‘acceptable’ experiences as an intern. Perceptions of training were measured as ‘positive’, ‘negative’, or ‘unknown’. Feelings of emotional exhaustion (burnout) and depersonalisation (callousness) retained their 5-point scale.

## Results

### Response rate

**Fig. 1:**
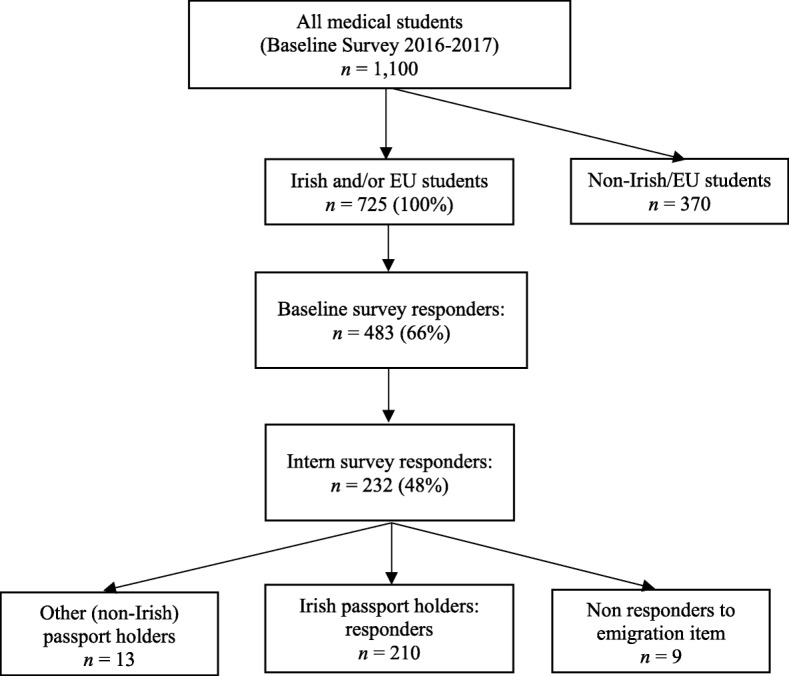
Overview of sample

The baseline survey was sent in late 2016 to 1100 final year medical students in all six Irish medical schools. The target sample was 725 Irish/European Union students, eligible to work in Ireland as interns following graduation [30]. Completed surveys were returned by 66% of the sample (*n* = 483) (see Fig. 1). Of those, 232 responded to the intern sweep in June 2018, giving a response rate for the intern sweep of 48%.

As the primary outcome of interest was to examine factors associated with the emigration of Irish doctors from Ireland (and not the migration plans of non-nationals, even if they had graduated from Irish medical schools), non-Irish passport holders (*n* = 13) and those who did not respond to the item on migration (*n* = 9) were excluded from further analysis, providing a final sample size of *n* = 210. While the response rate of 48% (32% of the original cohort) was adequate, the sample size was only sufficient to identify factors associated with intention to migrate and was not sufficient to systematically examine interactions between all predictor variables.

### Sample characteristics by intention to migrate

Table 1 shows the profile of Irish passport holders across the categories of intention to migrate. All (*n* = 210) had worked over the previous year as an intern in Ireland and were therefore eligible to continue their training in Ireland. Average age of the interns was 26 years (IQR 24–27). Route to study medicine was primarily through DEM (*n* = 142, 68%), with 68 (32%) entering via the GEM programme. The most popular first choice specialty was Medicine (36%), followed by Surgery (31%) and General Practice (13%).

**Table 1:** Profile by categories of intention to migrate

	Remain in Ireland	Leave but return	Leave permanently	Total	Statistics
	*n* (%)	*n* (%)	*n* (%)	*n* (%)	*p*
Total	76 (36)	119 (57)	15 (7)	210 (100)	
Age (Intern)					
≤ 26 years	32 (24)	93 (69)	10 (7)	135 (100)	< 0.00 1
27 years +	44 (60)	25 (34)	4 (5)	73 (100)	
Study pathway					
Direct Entry Medicine (DEM)	35 (25)	96 (68)	11 (8)	142 (100)	< 0.00 1
Graduate Entry Medicine (GEM)	41 (60)	23 (34)	4 (6)	68 (100)	
Sex					
Women	41 (37)	62 (55)	9 (8)	112 (100)	0.85 9
Men	35 (36)	56 (58)	6 (6)	97 (100)	
Amount of debt (€)					
0–4999	19 (35)	34 (62)	2 (4)	55 (100)	0.09 1^†^
5000–10 000	10 (24)	27 (66)	4 (10)	41 (100)	
10 001–20 000	8 (23)	25 (71)	2 (57)	35 (100)	
20 001–30 000	4 (36)	7 (64)	0 (0)	11 (100)	
30 001–40 000	4 (80)	1 (20)	0 (0)	5 (100)	
40 001–50 000	2 (66)	1 (33)	0 (0)	3 (100)	
50 001–60 000	6 (50)	5 (42)	1 (8)	12 (100)	
60 001–70 000	7 (58)	5 (42)	0 (0)	12 (100)	
70 001–80 000	5 (45)	5 (45)	1 (9)	11 (100)	
80 001–90 000	2 (50)	2 (50)	0 (0)	4 (100)	
90 001–100 000	0 (0)	0 (0)	1 (100)	1 (100)	
100 001–200 000	2 (50)	1 (25)	1 (25)	4 (100)	
200 000+	1 (33)	1 (33)	1 (33)	3 (100)	
Specialty choice					
Medicine	22 (29)	50 (67)	3 (4)	75 (100)	0.07 7^†^
Surgery	23 (36)	35 (55)	6 (9)	64 (100)	
General Practice	16 (59)	9 (33)	2 (7)	27 (100)	
Paediatrics	5 (36)	8 (57)	1 (7)	14 (100)	
Obstetrics/Gynaecology	4 (40)	6 (60)	0 (0)	10 (100)	
Psychiatry	5 (56)	4 (44)	0 (0)	9 (100)	
Other	1 (12)	5 (63)	2 (25)	8 (100)	
Not sure	0 (0)	2 (67)	1 (33)	3 (100)	

^†^Fisher’s exact test

Almost two thirds (64%) of the 210 respondents intended to go abroad to practise medicine, with 57% intending to return to Ireland to continue their medical career and 7% intending to leave Ireland permanently; 36% intended to remain in Ireland to practise medicine. Just over half the respondents had debts of over €10K (54%).

### Experiences as an intern

The respondents rated as negative their experience of the following while working as interns: staffing levels (71%), designated/protected training time (70%), and non-core task allocation (56%). Mentoring supports were rated negatively by 50% of respondents (Table 2).

**Table 2:** Thinking only about *your experience as an intern*, please rate the following factors within your training and working environment

	Negative	Acceptable	Positive	Total
	%	%	%	*n*
Staffing levels in my workplace	71	29	0	201
Designated/protected training time	70	29	1	201
Non-core task allocation*	56	39	4	201
Mentoring supports within my training programme	50	44	6	201
Level of supervision of my training	45	52	2	201
Level of preparedness on starting work as an intern	45	53	1	201
Level of stress in my working environment	40	60	0	201
Costs associated with training in my specialty	33	62	5	201
Level of bullying in the workplace	24	42	34	201

*Interns in Ireland are tasked with routine activities that in other countries are undertaken by other members of the hospital team such as nurses and technicians. Non-core activities include taking blood samples, inserting intravenous cannulas, giving first-dose drugs, and discharging patients

In response to the statement measuring depersonalization (‘I have become more callous toward people since I took this job’), over one third (35%) reported experiencing feelings of callousness once a week or more often in the previous 12 months. Responding to the measure of emotional exhaustion (‘I felt burned out from my work’), 30% reported feelings of burnout once a week or more often (Table 3).

**Table 3:** Prevalence of burnout and callousness in the year working as an intern

	Never	A few times in the past year	Once a month or less	A few times a month	Once a week	A few times a week	Every day	Total
	%	%	%	%	%	%	%	*n*
I felt burned out from my work	3	31	15	20	10	15	5	201
I have become more callous toward people since I took this job	10	24	12	19	13	12	10	201

### Perception of training in Ireland

Table 4 displays responses to a number of statements pertaining to respondents’ perceptions of postgraduate training in Ireland. For analysis purposes, positive, negative, and ‘don’t know’ responses (range 0–9) categorised the respondent into overall ‘positive perception’, overall ‘negative perception’, or ‘Don’t knows’. Almost all responders perceived work-life balance abroad to be better (94%), with a similar number being uncertain about securing attractive permanent posts in Ireland (91%). Respondents reported negative perceptions of post graduate training pathways (69%), service demands (non-core tasks) (68%), and opportunities for postgraduate training (67%). It was notable that many respondents had no clear expectation around training in Ireland, with 52% reporting no opinion as to whether appointment panels preferred specialists who had trained abroad, and 42% unaware whether supervision in postgraduate training schemes in Ireland was good or not.

**Table 4:** Considering *your perceptions of training in Ireland*, please rate the following 9 statements

	True	False	Don’t know	Total
	%	%	%	*n*
There are opportunities for a better work-life balance abroad	94	2	4	197
There is uncertainty about getting attractive permanent posts in Ireland	91	2	7	198
Training pathways/duration in Ireland are not predictable for NDHDs	69	19	12	198
Service tasks crowd out training opportunities for trainees	68	4	28	197
There are opportunities for better training abroad	67	4	29	196
The length of specialty training in Ireland is too long	32	44	24	197
Appointment panels prefer specialists who have trained abroad	30	17	52	197
There is adequate time for family/personal life as an NCHD	26	67	7	198
Supervision in postgraduate training schemes in Ireland is good	23	35	42	197

### Multinomial logistic regression analysis

Multinomial logistic regression analysis was undertaken (see Table 5). The comparison group for the two sets of leavers (those who intended to leave but return (*n* = 119) and those who intended to leave permanently (*n* = 15)) was the group of interns who intended to remain in Ireland (*n* = 76). Additionally, a chi-square (*χ*^*2*^) statistic test compared the results for each type of leaver.

**Table 5:** Multinomial logistic regression analysis of doctors intending to migrate (*n* = 134). Comparison group was *n* = 76 doctors intending to remain in Ireland. Unadjusted and adjusted for DEM/GEM

	Leave but return		Leave permanently			
	RRR [95% CI]	*p*	RRR [95% CI]	*p*	χ^2†^	*p*
Unadjusted						
Study pathway—GEM entry (v. DEM)	0.20 [0.11–0.39]	0.00 0	0.31 [0.09–1.06]	0.06 2	0.4	0.50 7
Adjusted for Study pathway						
Sex**—**male (v. female)	0.86 [0.47–1.60]	0.64 2	1.19 [0.38–3.74]	0.76 1	0.3	0.56 4
Age	0.88 [0.76–1.01]	0.07 8	0.82 [0.57–1.17]	0.27 2	0.2	0.69 1
Debt - €10 K+ (v. <€10 K)	1.01 [0.51–1.99]	0.97 7	1.18 [0.33–4.23]	0.80 2	0.1	0.80 6
Specialty (reference GP)						
Medicine	3.01 [1.09–8.34]	0.03 4	0.89 [0.13–6.13]	0.90 4		
Surgery	1.85 [0.66–5.22]	0.24 6	1.59 [0.27–9.32]	0.60 5		
Other	1.82 [0.60–5.56]	0.29 1	1.20 [0.17–8.60]	0.85 5		
Experience as an intern						
Overall negative experience	1.16 [1.00–1.34]	0.04 3	1.54 [1.15–2.04]	0.00 3	4.1	0.04 3
Emotional exhaustion (burnout)	1.08 [0.89–1.32]	0.41 0	1.57 [1.08–2.27]	0.01 7	4.1	0.04 2
Depersonalization (callousness)	1.23 [1.03–1.46]	0.02 3	1.77 [1.24–2.53]	0.00 2	4.3	0.03 7
Perception of training in Ireland						
Overall positive perception	0.94 [0.73–1.19]	0.58 9	0.50 [0.26–0.94]	0.03 2	3.9	0.04 8
Overall negative perception	1.16 [1.01–1.34]	0.03 7	1.15 [0.89–1.50]	0.28 4	0.0	0.95 2
Don't know	0.87 [0.72–1.05]	0.14 4	0.83 [0.58–1.20]	0.51 6	0.1	0.81 6

RRR relative risk ratio, CI confidence interval, GEM Graduate Entry Medicine, DEM Direct Entry Medicine, GP General Practice

^†^The reported chi-square test examines the relationship between the results of the two categories of leavers

Age and study pathways (DEM/GEM) were each significantly associated with intention to migrate (see Table 1) but were also strongly collinear (average age DEM = 25 (IQR 24–25); average age GEM = 29 years (IQR 27–31); *r* = 0.69, *p* < 0.001). When adjusted for study pathway, bivariable analysis for age was not significantly associated with intention to migrate (*p* = 0.09). However, when adjusted for age, study pathway remained a significant predictor (*p* = 0.03, data not shown). All other predictors of intention to migrate were therefore adjusted for study pathway (DEM/GEM) (Table 5).

Being a GEM graduate significantly reduced the likelihood of leaving to return (relative risk ratio (RRR) 0.20, 95% confidence interval (CI) (0.11–0.39), *p* < 0.001), while intention to specialise in Medicine (compared with General Practice) significantly increased the likelihood of leaving to return (RRR 3.01 (1.09–8.34), *p* < 0.034).

A negative experience as an intern significantly increased the likelihood of leaving to return (1.16 (1.00–1.34), *p* = 0.043), and also leaving permanently (1.54 (1.15–2.04), *p* = 0.003), with a significantly stronger association for leaving permanently *χ*^2^ (1) 4.1, *p* = 0.036. A positive perception of training in Ireland significantly reduced the likelihood of leaving permanently (0.50 (0.26–0.94), *p* = 0.032), while a negative perception of training in Ireland increased the likelihood of leaving to return (1.16 (1.01–1.34), *p* = 0.037). Emotional exhaustion (burnout) was associated with an increased likelihood of leaving permanently (1.57 (1.08–2.27), *p* = 0.017); depersonalisation (callousness) was associated with an increased likelihood of leaving and returning (1.23 (1.03–1.46), *p* = 0.023), with a stronger association with leaving permanently (1.77 (1.24–2.53), *p* = 0.002), *χ*^*2*^ (1) 4.3, *p* = 0.037.

## Discussion

Migration of doctors from their country of training impacts on medical workforce planning [1, 9, 21, 39], represents a major loss of state investment in medical education [3, 40], and may impact negatively on health sector goals. There is clear evidence that once abroad, increasing numbers of early-career doctors who emigrate with intentions of returning to their homeland to work never actually return [21, 25, 28, 29, 41]. The continued emigration of early-career doctors from Ireland is resulting in a loss of investment to the exchequer [13], necessitates high levels of replacement through inward migration of non-EU doctors (in 2017, 42% of doctors in Ireland were non-EU-trained [16, 22], and is contributing to the high number of Ireland’s currently unfilled consultant posts [41]. In addition, doctor emigration deprives the Irish health system of ‘potential leaders who might otherwise demand, initiate and deliver reform’ [20].

Despite implementation since early 2015 of a multi-stakeholder, Department of Health-led national strategy, designed to increase graduate retention in Ireland [42–44], this study of interns found that almost two thirds (64%) of our respondents intended to leave after their mandatory year of working within the Irish healthcare system. This should be of huge concern: while most intended (or hoped) to return, an earlier study of Irish-trained doctors abroad showed that many would not act on this intention unless working conditions and career opportunities in Ireland improved [23]. Furthermore, evidence shows that as emigrants’ roots abroad are established, emigration becomes permanent [25, 28].

Our study demonstrated an important and statistically significant predictor of the intention to migrate, which remained when adjusted for age, and which has not previously been reported: well over half (60%) of GEM doctors intended to remain in Ireland following their internship, compared with only one quarter (24%) of DEM doctors. This finding suggests that GEM doctors are more likely to stay working within the Irish health system. However, among the doctors who planned to leave (64%, *n* = 134), the same proportion of DEM and GEM doctors planned to return (89% DEM, 85% GEM) and to leave permanently (DEM 10%, GEM 15%) (*χ*^*2*^ (1) = 0.46, *p* = 0.504).

Independent of the study pathway, doctors’ intention to migrate were significantly associated with their negative working experiences, and possible effects of their experiences, during their mandatory year as an intern. This was seen most strongly for those intending to leave permanently. In this study, negative working experiences, as previously reported in research among doctors in postgraduate training programmes in Ireland [18] and reported by Irish-trained doctors abroad in relation to working in Ireland [23, 42], are found to be evident after as little as 1 year of work as a hospital doctor. Our study found that of the interns who responded to our survey, 70% rated as negative both ‘protected training time’ and the ‘staffing levels in their workplace’, while close to 60% rated as negative their experiences of ‘non-core task allocation’. While reported previously in more experienced hospital doctors [24, 45], this study also shows that burnout and callousness were common, experienced at least once a week, in one third of doctors after a single year working in Irish hospitals. These experiences were also presenting as predictors of intention to migrate (with the strongest effect for both factors seen in those intending to leave permanently); however, in the absence of a temporal relationship, no causal link with working conditions has been demonstrated, which may be a subject for future studies.

These findings should be of great concern to Irish health workforce policy makers. The 2014 national strategy was introduced specifically to address unsatisfactory training and working conditions in order to improve graduate retention [42, 43]. The recommendations targeted the enforcement of protected training time and the reduction of non-core task allocation for doctors in training. Our results show that these areas continue to be sources of difficulty for interns in 2018 and are contributing to interns’ migration intentions.

Negative perceptions of training in Ireland were significantly associated with interns’ intention to leave and return, while a positive perception reduced the likelihood of leaving permanently. Intended specialty choice was also a statistically significant factor, with those planning a career in Medicine more likely to leave and return compared with those planning to train in General Practice. Surprisingly, despite being at the point of making training and career choices, many respondents appeared not to have formed an opinion about the training landscape in Ireland (Table 4). This may indicate a necessity for training bodies to not only improve training options in Ireland, but also promote awareness of training options during the 1-year internship [43]. However, considering the global networks among medical graduates [23], it will be necessary for Ireland’s medical training to be viewed as competitive when considered against the training opportunities, staffing levels, and working conditions in destination countries such as Australia [21, 41].

While our findings are limited by the relatively small sample size, low response rates from medical professionals, and doctors in training, are well documented [46], with some published findings reporting response rates as low as 7.8% [47]. And while there is little known about the non-responders (sampling frames were unavailable from medical schools and/or hospitals), of those who did respond, a very high proportion completed the entire survey (96%). Our findings around burnout and callousness require further analysis in order to establish the direction of, and predisposing factors for, these outcomes and also to establish if they are a precursor to, or a subsequent development of, an intention to emigrate.

## Conclusion

With the high number of Irish doctors emigrating, and the consequent reliance on the dubious strategy of international recruitment to fill vacant posts [12], the strikingly different migration intentions of the GEM doctors compared to the DEM doctors represent important new evidence. Our findings suggest that by increasing the number of GEM training places, Ireland could potentially retain more of their domestically-trained doctors, slowing or stemming the outflow of early-career doctors. Indeed, with the phenomenon of early migration by medical graduates being experienced globally, similar-styled GEM programmes—recruiting older, more experienced, and possibly more resilient applicants to medical training—might be considered in other countries that are experiencing comparable patterns of doctor emigration [5, 6, 8]. However, for Ireland, as is clear also from our findings, any increase in GEM places must be in addition to—not as a substitute for—improving the working conditions and training opportunities of these expensively trained, internationally mobile, highly sought-after early-career doctors.

Experiences as an intern—the first year working as a doctor—may well contain the critical events that will determine the disposition of graduates toward ultimately making their careers in the country that trained them—regardless of whether they plan a period living and working abroad. This study points to the need for further research to explore the causes and consequences of burnout and other dimensions of ill-health in early-career doctors and to determine and understand what factors may be contributing to better retention intentions among GEM graduates.

## Data Availability

The datasets used and/or analysed during the current study are available from the corresponding author on reasonable request.

## Footnotes

1 Practising doctors are defined as providing direct care to patients and measured per 1000 inhabitants

## References

[1] Aluttis C, Bishaw T, Frank MW (2014). The workforce for health in a globalized context – global shortages and international migration. Glob Health Action..

[2] Scheffler RM, Arnold DR (2019). Projecting shortages and surpluses of doctors and nurses in the OECD: what looms ahead. Health Econ Policy Law..

[3] Jourdain A, Pham T (2017). Mobility of Physicians in Europe: Health Policies and Health Care Provision. Santé Publique..

[4] Hervey G. The EU exodus: When doctors and nurses follow the money [Internet]. POLITICO 2017 [cited 2019 Feb 27]. Available from: https://www.politico.eu/article/doctors-nurses-migration-health-care-crisis-workers-follow-the-money-european-commission-data/

[5] Lambert TW, Smith F, Goldacre MJ (2017). Why doctors consider leaving UK medicine: qualitative analysis of comments from questionnaire surveys three years after graduation. J R Soc Med..

[6] Ramos P, Alves H (2017). Migration intentions among Portuguese junior doctors: Results from a survey. Health Policy Amst Neth..

[7] Pantenburg B, Kitze K, Luppa M, König H-H, Riedel-Heller SG (2018). Physician emigration from Germany: insights from a survey in Saxony, Germany. BMC Health Serv Res..

[8] Suciu ŞM, Popescu CA, Ciumageanu MD, Buzoianu AD (2017). Physician migration at its roots: a study on the emigration preferences and plans among medical students in Romania. Hum Resour Health..

[9] Gauld R, Horsburgh S (2015). What motivates doctors to leave the UK NHS for a ‘life in the sun’ in New Zealand; and, once there, why don’t they stay?. Hum Resour Health..

[10] Teo WZW (2018). A closer look at the junior doctor crisis in the United Kingdom’s National Health Services: is emigration justifiable?. Camb Q Healthc Ethics..

[11] Bidwell P, Humphries N, Dicker P, Thomas S, Normand C, Brugha R (2013). The national and international implications of a decade of doctor migration in the Irish context. Health Policy..

[12] Brugha R, McAleese S, Dicker P, Tyrrell E, Thomas S, Normand C, et al. Passing through – reasons why migrant doctors in Ireland plan to stay, return home or migrate onwards to new destination countries. Hum Resour Health [Internet]. 2016 [cited 2019 Jan 8];14(S1). Available from: http://human-resources-health.biomedcentral.com/articles/10.1186/s12960-016-0121-z 10.1186/s12960-016-0121-z PMC4943478 27381409

[13] Campbell T (2015). Staff Paper 2015: Medical Workforce Analysis. Ireland and the European Union compared [Internet].

[14] Department of Health. Strategic review of medical training and career structure. Final report. [Internet]. Dublin; 2014. Available from: Http://Health.Gov.Ie/Wp-Content/Uploads/2014/07/SRMTCS_Final_Report_300614_FINAL1.Pdf.

[15] OECD (2019). Health at a Glance 2017: OECD Indicators [Internet].

[16] OECD. Health care resources: health workforce migration [Internet]. 2018 [cited 2019 Apr 15]. (Migration of doctors). Available from: https://stats.oecd.org/Index.aspx?QueryId=74639

[17] WHO (2010). Global code of practice on the international recruitment of health personnel.

[18] Clarke N, Crowe S, Humphries N, Conroy R, O’Hare S, Kavanagh P, et al. Factors influencing trainee doctor emigration in a high income country: a mixed methods study. Hum Resour Health [Internet]. 2017 [cited 2019 Jan 8];15(1). Available from: http://human-resources-health.biomedcentral.com/articles/10.1186/s12960-017-0239-7 10.1186/s12960-017-0239-7 PMC5611654 28942731

[19] Gorman D (2018). Matching the production of doctors with national needs. Med Educ..

[20] Humphries N, Crowe S, McDermott C, McAleese S, Brugha R. The consequences of Ireland’s culture of medical migration. Epidemiol Public Health Med Artic. 2017 Dec 28;87. 10.1186/s12960-017-0263-7 PMC5745907 29282076

[21] Humphries N, Crowe S, Brugha R. Failing to retain a new generation of doctors: qualitative insights from a high-income country. BMC Health Serv Res [Internet]. 2018 [cited 2019 Jan 8];18(1). Available from: https://bmchealthservres.biomedcentral.com/articles/10.1186/s12913-018-2927-y 10.1186/s12913-018-2927-y PMC5830046 29486756

[22] Brugha R, Cronin FM, Clarke N. Retaining our doctors. Medical Workforce Evidence, 2013-18 [Internet]. RCSI Health Workforce Research Group; 2018. Available from: http://www.healthworkforceireland.com/uploads/1/0/6/5/10659222/ad3310_retaining_our_doctors_responses.pdf

[23] Humphries N, McAleese S, Matthews A, Brugha R. ‘Emigration is a matter of self-preservation. The working conditions . . . are killing us slowly’: qualitative insights into health professional emigration from Ireland. Hum Resour Health [Internet]. 2015 [cited 2019 Jan 8];13(1). Available from: http://human-resources-health.biomedcentral.com/articles/10.1186/s12960-015-0022-6 10.1186/s12960-015-0022-6 PMC4437248 25981629

[24] Hayes B, Walsh G, Prihodova L. National study of wellbeing of hospital doctors in Ireland. [Internet]. Dublin: Royal College of Physicians of Ireland (RCPI); 2017 [cited 2019 Feb 26]. Available from: https://rcpi-live-cdn.s3.amazonaws.com/wp-content/uploads/2017/05/Wellbeing-Report-web.pdf

[25] Sharma A, Lambert TW, Goldacre MJ (2012). Why UK-trained doctors leave the UK: cross-sectional survey of doctors in New Zealand. J R Soc Med..

[26] Scharer S, Freitag A (2015). Physicians’ exodus: why medical graduates leave Austria or do not work in clinical practice. Wien Klin Wochenschr..

[27] Buchan J, Dubois C, McKee M, Nolte E. *Human resources for health in Europe*. European Observatory on Health Systems and Policies Series. Berkshire: Open University Press.

[28] McAleese S, Clyne B, Matthews A, Brugha R, Humphries N. Gone for good? An online survey of emigrant health professionals using Facebook as a recruitment tool. Hum Resour Health [internet]. 2016;14 Available from: 10.1186/s12960-016-0130-y. 10.1186/s12960-016-0130-y PMC4943487 27381189

[29] McDermott C, Sheridan M, Moore K, Gosbell A (2015). The medical boomerang: will it come back?. Emerg Med J..

[30] Department of Health & Children (2006). Fottrell Report. Medical education in Ireland – a new direction.

[31] Crowe S, Clarke N, Brugha R (2017). “You do not cross them”: hierarchy and emotion in doctors’ narratives of power relations in specialist training. Soc Sci Med..

[32] Cleland JA, Johnston PW, Anthony M, Khan N, Scott NW. A survey of factors influencing career preference in new-entrant and exiting medical students from four UK medical schools. BMC Med Educ [Internet]. 2014;14. Available from: 10.1186/1472-6920-14-151 10.1186/1472-6920-14-151 PMC4131477 25056270

[33] Querido SJ, Vergouw D, Wigersma L, Batenburg RS, De Rond MEJ, Ten Cate OTJ (2016). Dynamics of career choice among students in undergraduate medical courses. A BEME systematic review: BEME Guide No. 33. Med Teach..

[34] Medical Council (2018). Regional Inspection of Saolta University Health Care Group [Internet].

[35] West CP, Dyrbye LN, Sloan JA, Shanafelt TD (2009). Single item measures of emotional exhaustion and depersonalization are useful for assessing burnout in medical professionals. J Gen Intern Med..

[36] West CP, Dyrbye LN, Satele DV, Sloan JA, Shanafelt TD (2012). Concurrent validity of single-item measures of emotional exhaustion and depersonalization in burnout assessment. J Gen Intern Med..

[37] Cleland JA, Johnston PW, French FH, Needham G. Associations between medical school and career preferences in Year 1 medical students in Scotland. Med Educ [Internet]. 2012;46. Available from: 10.1111/j.1365-2923.2012.04218.x 10.1111/j.1365-2923.2012.04218.x 22515755

[38] Oireachtas H of the. Data Protection Act 2018 – No. 7 of 2018 – Houses of the Oireachtas [Internet]. 2018 [cited 2019 Mar 5]. Available from: https://www.oireachtas.ie/en/bills/bill/2018/10

[39] Medical Council. Medical Workforce Intelligence Report. A report on the 2016 and 2017 Annual Registration Retention & Voluntary Registration Withdrawal Surveys [Internet]. Dublin; 2019 [cited 2019 Apr 12]. Available from: https://www.medicalcouncil.ie

[40] Mills EJ, Kanters S, Hagopian A, Bansback N, Nachega J, Alberton M. The financial cost of doctors emigrating from sub-Saharan Africa: human capital analysis. BMJ [Internet]. 2011;343. Available from: 10.1136/bmj.d7031 10.1136/bmj.d7031 PMC3223532 22117056

[41] Humphries N, Connell J, Negin J, Buchan J (2019). Tracking the leavers: towards a better understanding of doctor migration from Ireland to Australia 2008–2018. Hum Resour Health..

[42] Department of Health. Strategic Review of Medical Training and Career Structure Final Report [Internet]. Dublin; 2014 [cited 2019 Mar 21]. Available from: https://health.gov.ie/wp-content/uploads/2014/07/SRMTCS_Final_Report_300614_FINAL1.pdf

[43] Department of Health. Strategic Review of Medical Training and Career Structure – Seventh Progress Report [Internet]. Dublin; 2018 [cited 2019 Mar 21]. Available from: https://health.gov.ie/blog/publications/strategic-review-of-medical-training-and-career-structure-seventh-progress-report/

[44] Department of Health. Strategic Review of Medical Training and Career Structure - Eighth Progress Report [Internet]. 2018 [cited 2019 Mar 21]. Available from: https://health.gov.ie/blog/publications/strategic-review-of-medical-training-and-career-structure-eighth-progress-report/

[45] Hayes B, Prihodova L, Walsh G, Doyle F, Doherty S (2017). What’s up doc? A national cross-sectional study of psychological wellbeing of hospital doctors in Ireland. BMJ Open..

[46] Grava-Gubins I, Scott S (2008). Effects of various methodologic strategies: survey response rates among Canadian physicians and physicians-in-training. Can Fam Physician Med Fam Can..

[47] Cloitre A, Duval X, Hoen B, Alla F, Lesclous P (2018). A nationwide survey of French dentists’ knowledge and implementation of current guidelines for antibiotic prophylaxis of infective endocarditis in patients with predisposing cardiac conditions. Oral Surg Oral Med Oral Pathol Oral Radiol..

